# Mutation analysis of *"Endoglin" *and *"Activin receptor-like kinase" *genes in German patients with hereditary hemorrhagic telangiectasia and the value of rapid genotyping using an allele-specific PCR-technique

**DOI:** 10.1186/1471-2350-10-53

**Published:** 2009-06-09

**Authors:** Haneen Sadick, Johanna Hage, Ulrich Goessler, Jens Stern-Straeter, Frank Riedel, Karl Hoermann, Peter Bugert

**Affiliations:** 1 Department of Otolaryngology, Head and Neck Surgery, University Hospital of Mannheim, 68135 Mannheim, Germany; 2 Institute of Transfusion Medicine and Immunology, Red Cross Blood Service of Baden-Württemberg-Hessen, University of Heidelberg, Medical Faculty of Mannheim, 68135 Mannheim, Germany

## Abstract

**Background:**

Hereditary hemorrhagic telangiectasia (HHT), also known as Rendu-Osler-Weber syndrome, is an autosomal dominant disorder which is clinically characterised by recurrent epistaxis, mucocutaneous telangiectasia and visceral arteriovenous malformations. Genetic linkage studies identified two genes primarily related to HHT: endoglin (*ENG*) on chromosome 9q33-34 and activin receptor-like kinase1 (*ACVRL1*) on chromosome 12q13. We have screened a total of 41 unselected German patients with the suspected diagnosis of HHT. Mutation analysis for the *ENG *and *ACVRL1 *genes in all patients was performed by PCR amplification. Sequences were then compared to the HHT database http://www.hhtmutation.org sequences of the *ENG *mRNA (accession no. BC014271.2) and the *ACVRL1 *mRNA (accession no. NM000020.1).

**Results:**

We identified 15 different mutations in 18 cases by direct sequencing. Among these mutations, one novel *ENG *mutation could be detected which has not yet been described in the literature before. The genotype-phenotype correlation was consistent with a higher frequency of pulmonary arteriovenous malformations in patients with *ENG *mutations than in patients with *ACVRL1 *mutations in our collective.

**Conclusion:**

For rapid genotyping of mutations and SNPs (single nucleotide polymorphisms) in *ENG *and *ACVRL1*, allele-specific PCR methods with sequence-specific primers (PCR-SSP) were established and their value analysed.

## Background

Hereditary hemorrhagic telangiectasia (HHT, MIM#18730), also known as Rendu-Osler-Weber syndrome, is an autosomal dominant disorder of the fibrovascular tissue. Worldwide, HHT has a prevalence of 1 in 10.000 individuals [1,2]. This multi-systemic angiogenic disorder is clinically characterized by severe and recurrent hemorrhages due to epistaxis, mucocutaneous telangiectasia and arteriovenous malformations. Vascular lesions are present in two forms, either as telangiectases on the skin as well as the inner lining of the mouth, nose and gastrointestinal tract (GI-tract) and/or as direct arteriovenous shunts most commonly seen in the lung (*P*AVM), brain (*C*AVM) and liver (*H*AVM) [3-9]. Electron microscopy studies on lesion biopsies revealed that dilated postcapillary venules connect directly to arterioles without intervening capillary bed [10,11]. As a result, these vascular lesions can be the cause for severe complications involving gastrointestinal bleedings, the risk of embolism, stroke and abscesses [12].

Till now, HHT can not be cured. The treatment of HHT patients remains symptomatic or has preventive character. The Curaçao criteria are in clinical use for diagnosis of HHT [13]. However, the clinical manifestations of HHT can be very heterogeneous, both between families and among members of the same family, making the diagnosis of the disease at times very difficult.

In the 90s, genetic linkage studies revealed the two major types of disease, HHT1 and HHT2, caused by mutations in the *ENG *(endoglin) and *ACVRL1 *genes. The first locus (HHT type 1) was mapped to chromosome 9q33-34 [14,15], where the 40 kb endoglin (*ENG*, OMIM 187300) was defined as the affected gene [16], also associated with a high prevalence of pulmonary arteriovenous malformations [17]. The second locus (HHT type 2) mapping to chromosome 12q was identified as the 15 kb activin receptor-like kinase-1 gene (*ACVRL1*, OMIM 600376) [18,19]. It is characterised by a lower incidence of pulmonary and cerebral arteriovenous malformations than HHT1, but has a higher incidence of liver manifestation [17,20]. The corresponding endoglin and ALK-1 proteins are specific endothelial receptors of the transforming growth factor beta superfamily essential for maintaining vascular integrity [21,22]. Many mutations have been identified in *ENG *and *ACVRL1 *genes and support the haploinsufficiency model for HHT [23]. In recent studies, two more genes have been implicated in HHT, which have not yet been identified, the HHT3 gene on chromosome 5 [24], and the HHT4 gene on chromosome 7 [25]. In addition, SMAD4 mutations mapping to chromosome 18 have been observed in patients with a combined syndrome of juvenile polyposis and HHT (JPHT) [26].

In this study, we report mutational analysis of the two main genes *ENG *and *ACVRL1 *in a German population affected by HHT. We describe a novel mutation in the *ENG *gene in HHT patients which has not been described before. Furthermore, we determine the value of allele-specific PCR for rapid genotyping.

## Methods

### Patients and controls

**Figure 1:**
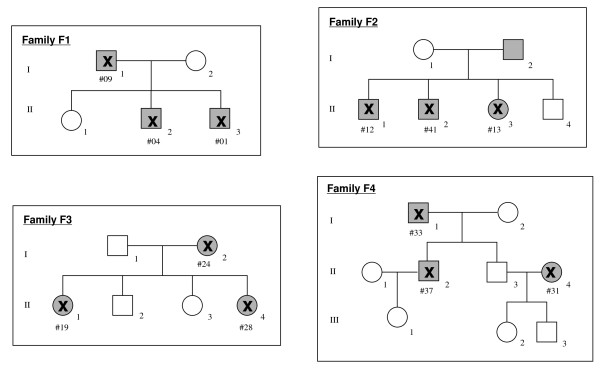
**Pedigree of the four small-sized HHT families**. White square and white circle = unaffected, grey square and grey circle = affected, X = DNA available, # = Pat.-no.

Anti-coagulated peripheral blood samples were obtained from 41 patients of German origin with tentative diagnosis of HHT at the Department of ORL, Head and Neck Surgery, University Hospital Mannheim, and from 768 healthy blood donors as a control group from the Institute of Transfusion Medicine and Immunology, Mannheim (Additional file 1). The HHT patients comprised 4 small-sized families (3 persons each) and 29 single cases (Fig. 1). Information on the clinical data including the medical history, physical examination and diagnostic screening was obtained in personal interviews with the affected individuals and from their patient records with all the data being documented in an HHT database. According to the Curaçao criteria, diagnosis for HHT included the presence of three out of four key symptoms: recurrent epistaxis, telangiectasia, hereditary with an affected first degree relative and visceral involvement [13] (Additional file 2). An informed consent was obtained from each individual and from parents of patients younger than 18 years according to the approval of the Ethic Committee of the University of Mannheim.

### Isolation of DNA

DNA was extracted from anti-coagulated peripheral blood samples using a commercial DNA isolation kit (QIAamp DNA blood mini kit; Qiagen, Hilden, Germany) according to the standard protocol.

### Sequencing of *ENG *and *ACVRL1 *exons

All coding exons and flanking intronic sequences of the *ENG *and *ACVRL1 *genes were PCR amplified using primers published previously [27,28]. PCR was performed in a total volume of 50 μl including 100 ng DNA, 0.5 μM each primer, 200 μM each dNTP, standard PCR buffer (Qiagen) and 1 U HotStar Taq DNA polymerase (Qiagen). The cycling parameters were as follows: initial denaturation for 15 min at 95°C; 35 cycles with 20 sec. at 94°C, 30 sec. at 60°C and 60 sec. at 72°C; final extension for 5 min at 72°C. After the PCR, products were run on 2% agarose gels. The 15 exons of *ENG *and the 9 exons of *ACVRL1 *were sequenced in 41 patients with HHT using Thermosequenase reagents (GE Healthcare Amersham), IRD700 and IRD800 labelled primers and an automated system (NEN^® ^Global IR^2 ^DNA Sequencer; LI-COR inc., Lincoln, NE, USA). Sequences were compared to the HHT database http://www.hhtmutation.org sequences of the *ENG *mRNA (accession no. BC014271.2) and the *ACVRL1 *mRNA (accession no. NM000020.1).

### PCR-SSP typing of SNPs

For rapid genotyping of the mutations and SNPs in *ENG *and *ACVRL1*, allele-specific PCR methods with sequence-specific primers (PCR-SSP) were established. Specific primer sequences and sizes of PCR products are given in Additional file 3. Part of the β-globin locus was amplified as an internal control PCR fragment (540 bp). According to a published PCR-SSP protocol [29,30], 20 ng DNA were subjected to 10 μl PCR reactions including 0.5 μM each allele-specific primers, 0.1 μM each internal control primer, standard PCR buffer (Qiagen), 200 μM each dNTP and 0.5 units Taq DNA polymerase (Qiagen). The cycling conditions were: 2 min initial denaturation at 94°C, followed by 10 cycles with 20 s denaturation at 94°C and 1 min annealing/extension at 65°C, followed by 20 cycles with 20 s duration at 94°C, 1 min annealing at 61°C and 30 s extension at 72°C. Amplification products were separated on 2% agarose gels containing 0.5 ng/ml ethidium bromide in a rapid agarose gel electrophoresis (RAGE; Cascade Biologics, Inc., Portland, OR, USA) chamber for 5 min at 25 V/cm. Results were obtained by visual inspection of the gels and were documented by using a UV documentation device with CCD camera (UVP, Inc., Upland, CA, USA).

## Results

The 15 coding exons of the *ENG *gene and the 9 exons of the *ACVRL1 *gene including flanking intron sequences were PCR amplified and sequenced from 41 patients with HHT. The sequence data were compared to each other and to the *ENG *and *ACVRL1 *sequences published in databases (HHT Mutation Database: http://www.hhtmutation.org). Mutations could be detected in 18 cases (43.9%).

### Sequence analysis of *ENG*

#### *ENG *mutations

**Figure 2:**
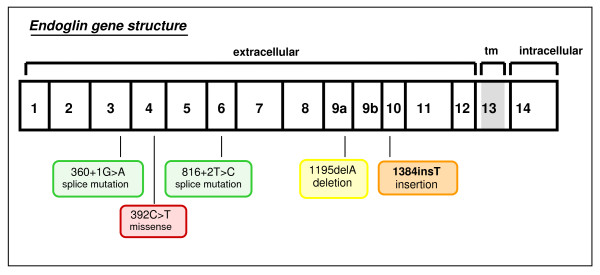
**Distribution of mutations in the *ENG *gene**. Novel mutation is given in bold.

We identified 5 different *ENG *mutations in 6 out of 41 HHT patients. The 5 different *ENG *mutations represented: 1 deletion, 1 insertion, 2 splice site mutations and 1 missense mutation (Additional file 4). In our study, mutation c.816+2T>C was detected in 2 related cases. One novel *ENG *mutation was detected in exon 10 (c.1384insT) causing a frameshift (p.Gln462fs) which has not yet been listed in the HHT gene database before. Altogether, all of the mutations found in *ENG *were detected in exons coding for the extracellular protein-domain. No mutations were detected in the region coding for the intracellular domain (Fig. 2).

#### Polymorphisms in the *ENG *gene

The 5 different polymorphisms and their relative frequencies identified in the *ENG *gene are presented in Additional file 5. A previously reported G to A substitution at position 207 of exon 2 (c.207G>A) was observed in 12 out of 41 HHT patients (29.3%). Two patients carried a c.1844C>T polymorphism in exon 13 (4.8%). At position 1029 (c.1029C>T) of exon 8, a C to T change was observed in 7 HHT patients (17%). A c.1347A>G polymorphism in exon 10 and a c.1771G>A polymorphism in exon 13 was observed in one HHT patient (2.4%).

### Sequence analysis of *ACVRL1*

#### *ACVRL1 *mutations

**Figure 3:**
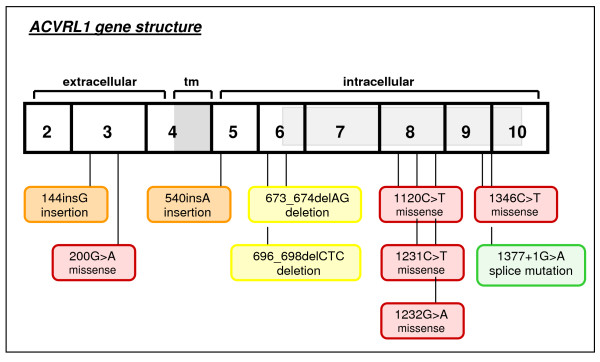
**Distribution of mutations in the *ACVRL1 *gene**.

Ten different *ACVRL1 *mutations could be identified in 12 out of 41 HHT patients, including 2 deletions, 2 insertions, 1 splice site mutation and 5 missense mutations (Additional file 6). The insertion c.144_145insG (p.Ala49fs) was detected in 2 members of the same family, whereas the missense mutation c.1120C>T (p.Arg374Trp) was detected in 2 unrelated cases. Half of the HHT2 associated *ACVRL1 *mutations consisted of single base substitutions, leading to amino acid changes. More than 75% of all *ACVRL1 *mutations were detected in exons coding for the intracellular domain (Fig. 3). Especially four out of five missense mutations (80%) were found in the receptor domain or the kinase domain, mainly affecting exon 8. No mutations were detected in the region coding for the transmembrane domain.

### PCR-SSP typing for *ENG *and *ACVRL1 *mutations

**Figure 4:**
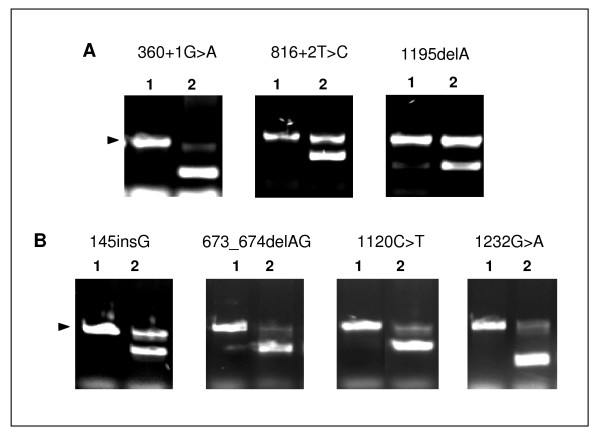
**Representative results from PCR-SSP analysis of different *ENG *(A) and *ACVRL1 *(B) mutations**. Three of the 5 PCR-SSP systems for *ENG *mutations and 4 of the 10 PCR-SSP systems for *ACVRL1 *mutations are shown as examples. The specificity of PCR-SSP for the corresponding mutations was given by the absence of the specific PCR product in healthy controls (each lane 1) and presence of the PCR product in the HHT patient (each lane 2). The internal control fragment of 540 bp in size (arrow head) was present in each reaction (or at least where the PCR-SSP product was absent).

Further investigation on allele and genotype frequencies focused on the mutations identified in our cohort of HHT patients. PCR-SSP systems could be established for all *ACVRL1 *mutations and all *ENG *mutations. A panel of DNA samples from HHT patients with different mutations was selected on the basis of the sequencing data and was used to validate all 15 PCR-SSP systems. The genotypes were estimated on the basis of the presence or the absence of the allele-specific PCR products (Fig. 4). All of the 41 HHT samples were retyped by PCR-SSP and data were compared to sequencing data. We could achieve 100% concordance of data, i.e. DNA samples with mutation revealed a positive PCR signal in the corresponding PCR-SSP system and DNA samples without mutation were negative in all 15 PCR-SSP systems. In addition, the 15 PCR-SSP systems were applied for genotyping of the *ENG *and *ACVRL1 *mutations in 768 healthy blood donor controls. None of the healthy control individuals showed an *ENG *or *ACVRL1 *mutation indicating the specificity of the gene variants for the HHT phenotype.

### Phenotype-Genotype Correlation

Diagnosis of HHT in an individual required that they met three or more of the following four Curaçao criteria: 1) spontaneous and recurrent epistaxis, 2) mucocutaneous telangiectasias, 3) organ manifestation with arteriovenous malformations (AVMs) including pulmonary, cerebral, hepatic, gastrointestinal and/or 4) hereditary with a first-degree relative with HHT [13]. All patients included in this study either met the criteria for manifest clinical diagnosis or suspected clinical diagnosis of HHT with at least three or two criteria present. Altogether, 71% of the patients (29 out of 41) had a manifest HHT diagnosis. All of the patients had as main clinical symptom recurrent epistaxis and a positive family history of HHT, followed by telangiectasias in 65.8% and AVMs of any type in 56% of the cases. Of the 41 patients tested, 18 patients were found to have sequence alterations, with 6 patients showing mutations for *ENG *and 12 patients revealing mutations for *ACVRL1*. The genotype-phenotype correlation of all HHT patients with confirmed mutation is given in Additional file 7.

## Discussion

Presently, *ENG *(endoglin) on chromosome 9q33-q34 and *ACVRL1 *(activin-receptor-like kinase) on chromosome 12q13 are the two genes primarily implicated in the development of Hereditary Hemorrhagic Telangiectasia (HHT). In literature, frequent mutations in both genes have been detected in different case studies indicating a common occurrence of *ENG *and *ACVRL1 *DNA changes in patients with HHT [27,28].

In a consecutive series of 41 patients, we performed clinical genetic testing for the two major types of disease, HHT1 and HHT2, caused by mutations in the *ENG *and *ACVRL1 *gene sequence for HHT. The series included 29 patients with clinical diagnosis of HHT who met three or more clinical features and 12 patients with suspected diagnosis with two clinical features according to the Curaçao criteria. The mutation detection rate in our series of 41 German patients was 44%. Based on the Curaçao criteria, 15 of the 18 patients (83%) with a mutation had a manifest clinical HHT diagnosis, with the remaining 3 patients (17%) fulfilling two criteria. This rate is similar to other studies which have been consistently described in literature [17,27,28]. Most of the mutations were found in the *ACVRL1 *gene with a total of 10 different mutations in 12 HHT patients whereas a total of 5 mutations were identified in the coding sequence of the *ENG *gene in 6 individuals. Most of the *ENG *mutations were widely distributed throughout the gene, not indicating any special hot spot areas within the gene. Out of five detected DNA changes, four mutations had been described previously as mutations in the HHT mutation database or recently been published [2,5,20,27,31-35]. The alterations included mainly nucleotide substitutions such as missense, splice site and frameshift mutations. In spite of the fact that only a small series of HHT patients was investigated in this study, one novel *ENG *mutation c.1384insT (p.Gln462fs) could be detected which had not been described before in the HHT mutation database. Unlike *ENG*, 50% of the mutations in the ACVRL1 gene were missense substitutions. Similar results had been published by Abdalla et al. who analysed all 123 till then known mutations of *ACVRL1*. In their study overview they could also register more than half (53%) of the mutations in the *ACVRL1 *gene as missense mutations [36,37]. As previously described in other HHT families, the frequency of *ACVRL1 *mutations was highest in exon 8 which might be an indicator for mutation hotspots. The other mutations c.144_145insG (p.Ala49fs), c.200G>A (p.Arg67Gln), c.540_541insA (p.Asp181fs), c.673_674delAG (p.Ser225fs), c.696_698delCTC (p.Ser233del), c.1346C>T (p.Pro449Leu) and c.1377+1G>A in *ACVRL1 *were already described by other workgroups, mostly located in the intracellular kinase domain of the gene [28,35,37-40]. Only two already known mutations could be detected in the extracellular domain of the gene. However, large deletions and insertions in *ENG *and *ACVRL1 *were not analyzed which could explain the low detection rate of mutations in our study compared to other studies described in literature [28,32,36].

As far as the genotype-phenotype correlation is concerned, most of the pulmonary arteriovenous malformations (*P*AVMs) in our study were detected in HHT patients with mutations for *ENG*. This phenomenon has already been described by previous studies given in literature, stating that mutations of *ENG *are mainly observed in HHT type 1 with an incidence of up to 40% for *P*AVMs, whereas mutations of *ALK1 *are primarily observed in HHT type 2 with an incidence of only 14% for *P*AVMs, which clinically distinguishes these two types of mutation [3,7,28,36]. Liver manifestations were more frequent in patients with *ACVRL1 *mutations, whereas manifestations of the skin and the GI-tract were seen equally distributed amongst HHT patients with *ENG *and *ACVRL1 *mutations.

Many studies report a mutation detection rate of up to 92% [27,28,33]. However, in our study we were able to identify only 43.9% of disease-causing mutations in a series of 41 HHT patients. The difference in sensitivity may result from several factors, including a very heterogeneous cohort of patients in terms of locations and initial physician involved, and because patients with only 2 Curaçao criteria were included in the study. In addition, only *ENG *and *ACVRL1 *genes were screened for mutations, but in some cases, mutations in SMAD4, HHT3 and HHT4 loci may be responsible for the clinical symptoms [24-26].

In daily clinical routine, the diagnosis of HHT is primarily based on the clinical findings according to the Curaçao criteria. Nevertheless, in many cases clinical diagnostic is at times very difficult due to the heterogeneous outcome and manifestation of the disease. This fact asks for additional diagnostic tools which facilitate the daily clinical work. Already in the past, many studies could show that the molecular characterization of HHT enables to predict the diagnosis before the manifestation of clinical symptoms. The results of the study reflect the very high and heterogenic distribution and allocation of mutations in HHT. With an allele-specific PCR technique (PCR-SSP) we could establish a diagnostic testing device for rapid genotyping of mutations. The exon sequencing of *ENG *and *ACVRL1 *confirmed the previously described polymorphisms and outlined one novel mutation Gln462fs in exon 10 of *ENG *gene. The PCR-SSP technique can easily be performed with standard laboratory equipment and may help to minimize costs for genetic testing in the routine diagnosis of family members of HHT patients with known mutations.

## Conclusion

In summary, this study is a further contribution to the determination of already known *ENG *and *ACVRL1 *mutations as well as the detection of one novel *ENG *mutation in HHT. Next to the Curaçao criteria, genetic analysis certainly contributes as an essential tool to a reliable diagnosis of clinically affected HHT patients and clinically un-symptomatic HHT patients, thus helping to take early preventive measures even before the occurrence of first clinical symptoms. The PCR-SSP technique could facilitate this high task of genetic analysis in routine HHT diagnostics and underlines the importance of using molecular diagnosis for early identification of individuals carrying mutations and being at risk of vascular complications.

## Competing interests

The authors declare that they have no competing interests.

## Authors' contributions

All the authors have read and approved the final manuscript. A large part of the mutational analysis was done by HS, JH under the supervision of PB. The rest of the mutational analysis was done by UG, JS, KH completing the analysis of mutations. Bank data collection, writing of the manuscript and the direction of all the work was done under HS, the main author.

## Pre-publication history

The pre-publication history for this paper can be accessed here:

http://www.biomedcentral.com/1471-2350/10/53/prepub

## Supplementary Material

Additional file 1 **Table 1**. Overview on the detailed clinical data of all HHT patients. Click here for file

Additional file 2 **Table 2**. Diagnostic criteria according to the Curaçao criteria for the diagnosis of HHT. Click here for file

Additional file 3 **Table 3**. Primers used in PCR-SSP genotyping for mutations in the *ENG *and *ACVRL1 *genes. Click here for file

Additional file 4 **Table 4**. Summary of mutations identified in the *ENG *gene. Click here for file

Additional file 5 **Table 5**. Polymorphisms in *ENG*. Click here for file

Additional file 6 **Table 6**. Summary of mutations identified in the *ACVRL1 *gene. Click here for file

Additional file 7 **Table 7**. Genotype-phenotype correlation in HHT patients with confirmed mutation. Click here for file

## References

[B1] Guttmacher AE Marchuk DA White RI Jr Hereditary hemorrhagic telangiectasia N Engl J Med 1995 333 14 918 24 10.1056/NEJM199510053331407 7666879

[B2] Abdalla SA Cymerman U Rushlow D Chen N Stoeber GP Lemire EG Letarte M Novel mutations and polymorphisms in genes causing hereditary hemorrhagic telangiectasia Hum Mutat 2005 25 3 320 1 10.1002/humu.9312 15712271

[B3] Kjeldsen A Kjeldsen J Gastrointestinal bleeding in patients with hereditary hemorrhagic telangiectasia Am J Gastroenterol 2000 95 415 418 10.1111/j.1572-0241.2000.01792.x 10685743

[B4] Reilly PJ Nostrant TT Clinical manifestations of hereditary hemorrhagic telangiectasia Am J Gastroenterol 1984 79 363 367 6609633

[B5] Kjeldsen AD Oxhøj H Andersen PE Green A Vase P Prevalence of pulmonary arteriovenous malformations (PAVMs) and occurrence of neurologic symptoms in patients with hereditary haemorrhagic telangiectasia (HHT) J Int Med 2000 248 255 262 10.1046/j.1365-2796.2000.00725.x 10971793

[B6] Shovlin CL Letarte M Hereditary haemorrhagic telangiectasia and pulmonary arteriovenous malformations: issues in clinical management and review of pathogenic mechanisms Thorax 1999 54 714 729 10413726 10.1136/thx.54.8.714 PMC1745557

[B7] Bernard G Mion F Henry L Plauchu H Paliard P Hepatic involvement in hereditary haemorrhagic telangiectasia: clinical, radiological, and hemodynamic studies of 11 cases Gastroenterology 1993 105 482 487 8335203 10.1016/0016-5085(93)90723-p

[B8] Garcia-Tsao G Korzenik JR Young L Henderson KJ Jain D Byrd B Pollak JS White RI Jr Liver disease in patients with hereditary hemorrhagic telangiectasia N Engl J Med 2000 343 13 931 936 10.1056/NEJM200009283431305 11006369

[B9] Sadick H Sadick M Götte K Naim R Riedel F Bran G Hörmann K Hereditary hemorrhagic telangiectasia: an update on clinical manifestations and diagnostic measures Wien Klin Wochenschr 2006 118 3–4 72 80 10.1007/s00508-006-0561-x 16703249

[B10] Braverman IM Keh A Jacobson BS Ultrastructure and three-dimensional organization of the teleangiectases of hereditary hemorrhagic teleangiectasia J Invest Dermatol 1990 95 422 427 10.1111/1523-1747.ep12555569 2212727

[B11] Jahnke V Ultrastructure of hereditary hemorrhagic telangiectasia Arch Otolaryngol Head and Neck Surg 1970 91 262 265 4189954

[B12] White RI Jr Pulmonary arteriovenous malformations: how do we diagnose them and why is it important to do so? Radiology 1992 182 3 633 5 1535872 10.1148/radiology.182.3.1535872

[B13] Shovlin CL Guttmacher AE Buscarini E Faughnan ME Hyland RH Westermann CJ Kjeldsen AD Plauchu H Diagnostic criteria for hereditary hemorrhagic telangiectasia (Rendu-Osler-Weber syndrome) Am J Med Genet 2000 91 1 66 7 10.1002/(SICI)1096-8628(20000306)91:1<66::AID-AJMG12>3.0.CO;2-P 10751092

[B14] McDonald MT Papenberg KA Ghosh S Glatfelter AA Biesecker BB Helmbold EA Markel DS Zolotor A McKinnon WC Vanderstoep JL Jackson CE Iannuzzi M Collins FS Boehnke M Porteous ME Guttmacher AE Marchuk DA A disease locus for hereditary haemorrhagic telangiectasia maps to chromosome 9q33-34 Nat Genet 1994 6 2 197 204 10.1038/ng0294-197 8162075

[B15] Shovlin CL Hughes JM Tuddenham EG Temperley I Perembelon YF Scott J Seidman CE Seidman JG A gene for hereditary haemorrhagic telangiectasia maps to chromosome 9q3 Nat Genet 1994 6 2 205 9 10.1038/ng0294-205 8162076

[B16] McAllister KA Grogg KM Johnson DW Gallione CJ Baldwin MA Jackson CE Helmbold EA Markel DS McKinnon WC Murrell J Endoglin, a TGF-beta binding protein of endothelial cells, is the gene for hereditary haemorrhagic telangiectasia type 1 Nat Genet 1994 8 4 345 51 10.1038/ng1294-345 7894484

[B17] Letteboer TG Mager JJ Snijder RJ Koeleman BP Lindhout D Ploos van Amstel JK Westermann CJ Genotype-phenotype relationship in hereditary haemorrhagic telangiectasia J Med Genet 2006 43 4 371 7 16155196 10.1136/jmg.2005.035451 PMC2563220

[B18] Berg JN Gallione CJ Stenzel TT Johnson DW Allen WP Schwartz CE Jackson CE Porteous ME Marchuk DA The activin receptor-like kinase 1 gene genomic structure and mutations in hereditary hemorrhagic telangiectasia type 2 Am J Hum Genet 1997 61 1 60 7 9245985 10.1086/513903 PMC1715857

[B19] Johnson DW Berg JN Baldwin MA Gallione CJ Marondel I Yoon SJ Stenzel TT Speer M Pericak-Vance MA Diamond A Guttmacher AE Jackson CE Attisano L Kucherlapati R Porteous ME Marchuk DA Mutations in the activin receptor-like kinase 1 gene in hereditary haemorrhagic telangiectasia type 2 Nat Genet 1996 13 189 95 10.1038/ng0696-189 8640225

[B20] Kuehl HK Caselitz M Hasenkamp S Wagner S El-Harith el-HA Manns MP Stuhrmann M Hepatic manifestation is associated with ALK1 in hereditary hemorrhagic telangiectasia: identification of five novel ALK1 and one novel ENG mutations Hum Mutat 2005 25 3 320 10.1002/humu.9311 15712270

[B21] Arthur HM Ure J Smith AJ Renforth G Wilson DI Torsney E Charlton R Parums DV Jowett T Marchuk DA Burn J Diamond AG Endoglin, an ancillary TGFbeta receptor, is required for extraembryonic angiogenesis and plays a key role in heart development Dev Biol 2000 217 42 53 10.1006/dbio.1999.9534 10625534

[B22] Oh SP Seki T Goss KA Imamura T Yi Y Donahoe PK Li L Miyazono K ten Dijke P Kim S Li E Activin receptor-like kinase 1 modulates transforming growth factor-beta 1 signaling in the regulation of angiogenesis Proc Natl Acad Sci USA 2000 97 2626 31 10716993 10.1073/pnas.97.6.2626 PMC15979

[B23] Pece-Barbara N Cymerman U Vera S Marchuk DA Letarte M Expression analysis of four endoglin missense mutations suggests that haploinsufficiency is the predominant mechanism for hereditary hemorrhagic telangiectasia type 1 Hum Mol Genet 1999 8 12 2171 81 10.1093/hmg/8.12.2171 10545596

[B24] Cole SG Begbie ME Wallace GM Shovlin CL A new locus for hereditary haemorrhagic telangiectasia (HHT3) maps to chromosome 5 J Med Genet 2005 42 7 577 82 15994879 10.1136/jmg.2004.028712 PMC1736109

[B25] Bayrak-Toydemir P McDonald J Akarsu N Toydemir RM Calderon F Tuncali T Tang W Miller F Mao R A fourth locus for hereditary hemorrhagic telangiectasia maps to chromosome 7 Am J Med Genet A 2006 140 2155 62 16969873 10.1002/ajmg.a.31450

[B26] Gallione CJ Repetto GM Legius E Rustgi AK Schelley SL Tejpar S Mitchell G Drouin E Westermann CJ Marchuk DA A combined syndrome of juvenile polyposis and hereditary haemorrhagic telangiectasia associated with mutations in MADH4 (SMAD4) Lancet 2004 363 852 9 10.1016/S0140-6736(04)15732-2 15031030

[B27] Abdalla SA Letarte M Hereditary haemorrhagic telangiectasia: current views on genetics and mechanisms of disease J Med Genet 2006 43 2 97 110 15879500 10.1136/jmg.2005.030833 PMC2603035

[B28] Schulte C Geisthoff U Lux A Kupka S Zenner HP Blin N Pfister M High frequency of ENG and ALK1/ACVRL1 mutations in German HHT patients Hum Mutat 2005 25 6 595 10.1002/humu.9345 15880681

[B29] Bugert P Hoffmann MM Winkelmann BR Vosberg M Jahn J Entelmann M Katus HA März W Mansmann U Boehm BO Goerg S Klüter H The variable number of tandem repeat polymorphism in the P-selectin glycoprotein ligand-1 gene is not associated with coronary heart disease J Mol Med 2003 81 8 495 501 10.1007/s00109-003-0459-2 12879153

[B30] Bugert P Lese A Meckies J Zieger W Eichler H Klüter H Optimized sensitivity of allele-specific PCR for prenatal typing of human platelet alloantigen single nucleotide polymorphisms Biotechniques 2003 35 1 170 4 12866418 10.2144/03351md05

[B31] Bossler AD Richards J George C Godmilow L Ganguly A Novel mutations in ENG and ACVRL1 identified in a series of 200 individuals undergoing clinical genetic testing for hereditary hemorrhagic telangiectasia HHT): correlation of genotype with phenotype Hum Mutat 2006 27 7 667 75 10.1002/humu.20342 16752392

[B32] Lesca G Burnichon N Raux G Tosi M Pinson S Marion MJ Babin E Gilbert-Dussardier B Rivière S Goizet C Faivre L Plauchu H Frébourg T Calender A Giraud S French Rendu-Osler Network Distribution of ENG and ACVRL1 (ALK1) mutations in French HHT patients Hum Mutat 2006 27 6 598 10.1002/humu.9421 16705692

[B33] Lesca G Olivieri C Burnichon N Pagella F Carette MF Gilbert-Dussardier B Goizet C Roume J Rabilloud M Saurin JC Cottin V Honnorat J Coulet F Giraud S Calender A Danesino C Buscarini E Plauchu H French-Italian-Rendu-Osler Network Genotype-phenotype correlations in hereditary hemorrhagic telangiectasia: data from the French-Italian HHT network Genet Med 2007 9 1 14 22 10.1097/GIM.0b013e31802d8373 17224686

[B34] Fernandez-L A Sanz-Rodriguez F Zarrabeitia R Perez-Molino A Morales C Restrepo CM Ramirez JR Coto E Lenato GM Bernabeu C Botella LM Mutation study of Spanish patients with hereditary hemorrhagic telangiectasia and expression analysis of Endoglin and ALK1 Hum Mutat 2006 27 3 295 10.1002/humu.9413 16470589

[B35] Fontalba A Fernandez-L A García-Alegria E Albiñana V Garrido-Martin EM Blanco FJ Zarrabeitia R Perez-Molino A Bernabeu-Herrero ME Ojeda ML Fernandez-Luna JL Bernabeu C Botella LM Mutation study of Spanish patients with hereditary hemorrhagic telangiectasia BMC Med Genet 2008 9 75 18673552 10.1186/1471-2350-9-75 PMC2518546

[B36] Wehner LE Folz BJ Argyriou L Twelkemeyer S Teske U Geisthoff UW Werner JA Engel W Nayernia K Mutation analysis in hereditary haemorrhagic telangiectasia in Germany reveals 11 novel ENG and 12 novel ACVRL1/ALK1 mutations Clin Genet 2006 69 3 239 45 10.1111/j.1399-0004.2006.00574.x 16542389

[B37] Abdalla SA Letarte M Hereditary haemorrhagic telangiectasia: current views on genetics and mechanisms of disease J Med Genet 2006 43 2 97 110 15879500 10.1136/jmg.2005.030833 PMC2603035

[B38] Klaus DJ Gallione CJ Anthony K Yeh EY Yu J Lux A Johnson DW Marchuk DA Novel missense and frameshift mutations in the activin receptor-like kinase-1 gene in hereditary hemorrhagic telangiectasia. Mutations in brief no. 164 Hum Mutat 1998 12 2 137 10.1002/(SICI)1098-1004(1998)12:2<137::AID-HUMU15>3.0.CO;2-M 10694922

[B39] Abdalla SA Cymerman U Johnson RM Deber CM Letarte M Disease-associated mutations in conserved residues of ALK-1 kinase domain Eur J Hum Genet 2003 11 4 279 87 10.1038/sj.ejhg.5200919 12700602

[B40] Lesca G Plauchu H Coulet F Lefebvre S Plessis G Odent S Rivière S Leheup B Goizet C Carette MF Cordier JF Pinson S Soubrier F Calender A Giraud S French Rendu-Osler Network Molecular screening of ALK1/ACVRL1 and ENG genes in hereditary hemorrhagic telangiectasia in France Hum Mutat 2004 23 4 289 99 10.1002/humu.20017 15024723

