# Supplementary material for: Mutation analysis of "Endoglin" and "Activin receptor-like kinase" genes in German patients with hereditary hemorrhagic telangiectasia and the value of rapid genotyping using an allele-specific PCR-technique
Source: BMC Med Genet. 2009 Jun 9;10:53. doi: 10.1186/1471-2350-10-53 (PMC2701415; doi:10.1186/1471-2350-10-53)
Supplement: Additional file 1 — Table 1. Overview on the detailed clinical data of all HHT patients. [file 1471-2350-10-53-S1.doc]

# Patient Sex Age Heredity Epistaxis Telangiectasia Organ manifestation Curacao

**(y) criteria**

#01 F^1^ f 69 x x ---- ---- 2

-----------------------------------------------------------------------------------------------------------------------------------------------

#02 S m 75 x x x GI-tract 4

-----------------------------------------------------------------------------------------------------------------------------------------------

#03 S f 59 x x x GI-tract 3

-----------------------------------------------------------------------------------------------------------------------------------------------

#04 F^1^ f 61 x x ---- ---- 2

-----------------------------------------------------------------------------------------------------------------------------------------------

#05 S f 78 x x x GI-tract, liver 4

-----------------------------------------------------------------------------------------------------------------------------------------------

#06 S f 88 x x x ---- 3

-----------------------------------------------------------------------------------------------------------------------------------------------

#07 S m 76 x x x GI-tract, liver 4

-----------------------------------------------------------------------------------------------------------------------------------------------

#08 S m 38 x x ---- ---- 2

-----------------------------------------------------------------------------------------------------------------------------------------------

#09 F^1^ f 85 x x x ---- 3

-----------------------------------------------------------------------------------------------------------------------------------------------

#10 S m 67 x x ---- liver 3

-----------------------------------------------------------------------------------------------------------------------------------------------

#11 S f 72 x x x GI-tract, lung 4

-----------------------------------------------------------------------------------------------------------------------------------------------

#12 F^2^ f 70 x x x GI-tract, liver, lung 4

-----------------------------------------------------------------------------------------------------------------------------------------------

#13 F^2^ m 68 x x ---- ---- 2

-----------------------------------------------------------------------------------------------------------------------------------------------

#14 S f 84 x x x liver 4

-----------------------------------------------------------------------------------------------------------------------------------------------

#15 S f 37 x x ---- ---- 2

-----------------------------------------------------------------------------------------------------------------------------------------------

#16 S f 72 x x x GI-tract, liver 4

-----------------------------------------------------------------------------------------------------------------------------------------------

#17 S m 38 x x ---- ---- 2

-----------------------------------------------------------------------------------------------------------------------------------------------

#18 S m 43 x x x ---- 3

-----------------------------------------------------------------------------------------------------------------------------------------------

#19 F^3^ m 44 x x x lung, brain 4

-----------------------------------------------------------------------------------------------------------------------------------------------

#20 S f 61 x x x GI-tract, liver, lung 4

-----------------------------------------------------------------------------------------------------------------------------------------------

#21 S m 79 x x x lung 4

-----------------------------------------------------------------------------------------------------------------------------------------------

#22 S f 73 x x x GI-tract 4

-----------------------------------------------------------------------------------------------------------------------------------------------

#23 S f 35 x x ---- ---- 2

-----------------------------------------------------------------------------------------------------------------------------------------------

#24 F^3^  m 76 x x x lung 4

-- ---------------------------------------------------------------------------------------------------------------------------------------------

#25 S f 87 x x x GI-tract 3

-----------------------------------------------------------------------------------------------------------------------------------------------

#26 S f 54 x x x ---- 3

-----------------------------------------------------------------------------------------------------------------------------------------------

#27 S f 57 x x x GI-tract, liver 4

-----------------------------------------------------------------------------------------------------------------------------------------------

#28 F^3^ m 51 x x x GI-tract, lung 4

-----------------------------------------------------------------------------------------------------------------------------------------------

#29 S m 68 x x ---- ---- 2

-----------------------------------------------------------------------------------------------------------------------------------------------

#30 S m 47 x x x lung 4

-----------------------------------------------------------------------------------------------------------------------------------------------

#31 F^4^ m 38 x x ---- ---- 2

-----------------------------------------------------------------------------------------------------------------------------------------------

#32 S f 70 x x ---- ---- 2

-----------------------------------------------------------------------------------------------------------------------------------------------

#33 F^4^ f 62 x x x GI-tract 4

--------------------------------------- -------------------------------------------------------------------------------------------------------

#34 S m 67 x x ---- ---- 2

-----------------------------------------------------------------------------------------------------------------------------------------------

#35 S m 65 x x x GI-tract, lung 4

-----------------------------------------------------------------------------------------------------------------------------------------------

#36 S m 88 x x x ---- 3

-----------------------------------------------------------------------------------------------------------------------------------------------

#37 F^4^ f 33 x x ---- ---- 2

-----------------------------------------------------------------------------------------------------------------------------------------------

#38 S m 71 x x ---- GI-tract, liver 3

-----------------------------------------------------------------------------------------------------------------------------------------------

#39 S m 81 x x x GI-tract, liver, lung 4

-----------------------------------------------------------------------------------------------------------------------------------------------

#40 S m 49 x x x ---- 3

-----------------------------------------------------------------------------------------------------------------------------------------------

#41 F^2^ f 70 x x x GI-tract 3

­­­­­­­­­­­­­­­­­­­­

Legend:

S = single cases, F^1-4^ = small-sized family cases with 3 persons each; f = female, m = male, y = years, x = present

Curacao criteria: hereditary, epistaxis, telangiectasia, organ manifestation

(<2 criteria: HHT probable, >3 criteria: HHT manifest)
