# Supplementary material for: Mutation analysis of "Endoglin" and "Activin receptor-like kinase" genes in German patients with hereditary hemorrhagic telangiectasia and the value of rapid genotyping using an allele-specific PCR-technique
Source: BMC Med Genet. 2009 Jun 9;10:53. doi: 10.1186/1471-2350-10-53 (PMC2701415; doi:10.1186/1471-2350-10-53)
Supplement: Additional file 2 — Table 2. Diagnostic criteria according to the Curaçao criteria for the diagnosis of HHT. [file 1471-2350-10-53-S2.doc]

**Table 2. Diagnostic criteria according to the Curaçao criteria for the diagnosis of HHT.** The diagnosis is definitive if 3 criteria are present, possible or suspected if 2 criteria are present, and unlikely if fewer than 2 criteria are present.

Epistaxis Spontaneous

Telangiectasia multiple, at characteristic sites: lips, oral cavity, fingers, nose

Visceral lesions GI-tract, *P*AVM, hepatic AVM, cerebral AVM, spinal AVM

Hereditary a first-degree relative with HHT according to these criteria
