# Supplementary material for: Mutation analysis of "Endoglin" and "Activin receptor-like kinase" genes in German patients with hereditary hemorrhagic telangiectasia and the value of rapid genotyping using an allele-specific PCR-technique
Source: BMC Med Genet. 2009 Jun 9;10:53. doi: 10.1186/1471-2350-10-53 (PMC2701415; doi:10.1186/1471-2350-10-53)
Supplement: Additional file 3 — Table 3. Primers used in PCR-SSP genotyping for mutations in the ENG and ACVRL1 genes. [file 1471-2350-10-53-S3.doc]

# **Table 3. Primers used in PCR-SSP genotyping for mutations in the *ENG* and *ACVRL1* genes.**^1^

| **PCR-SSP specificity** | **Primer sequences 5’-3’ (F: forward; R: reverse)** | **Location** | **PCR product  size (bp)** |
| --- | --- | --- | --- |
| ALK 145**insG** | F: CTACCTGCCGGGGGGGC R: CCCTTTATTGGCCAGAGCATGAG | Exon 3 Intron 3 | 276 |
| ALK 200G>**A** | F: GGCACCCCCAGGAACATCA R: CCCTTTATTGGCCAGAGCATGAG | Exon 3 Intron 3 | 224 |
| ALK 541**insA** | F: CCAGGACCTCCTGGACAGTA R: AGTAGGAGAAAGCGACTGTG | Exon 5 Intron 5 | 195 |
| ALK 674**delAG** | F: GCTTGTGGCACGGTGAGTG R: AAGAGGTTGATGCTGCAGGTGG | Exon 6 Intron 6 | 244 |
| ALK 698**delCTC** | F: GGCCGTCAAGATCTTCTCGAG R: AAGAGGTTGATGCTGCAGGTGG | Exon 6 Intron 6 | 222 |
| ALK 1120C>**T** | F: CCGAGAGTGGGCACCAAGT R: TTGCAGAGGGACGTGACTTG | Exon 8 Intron 8 | 256 |
| ALK 1231C>**T** | F: CTGTGGGAGATTGCCCGCT R: TTGCAGAGGGACGTGACTTG | Exon 8 Intron 8 | 145 |
| ALK 1232G>**A** | F: TGTGGGAGATTGCCCGCCA R: TTGCAGAGGGACGTGACTTG | Exon 8 Intron 8 | 144 |
| ALK 1346C>**T** | F: GTGTGGATCAGCAGACCCT R: GCATGGGCCAGGGTTGAAAG | Exon 9 Intron 9 | 213 |
| ALK 1377+1G>**A** | F: GGCTGGCTGCAGACCCGA R: GCATGGGCCAGGGTTGAAAG | Exon 9 Intron 9 | 188 |
| ENG 360+1G>**A** | F: ATCCCACTGCACTTGGCCTACA R: CAGGACCCTGGTGAATAATG | Exon 3 Intron 3 | 163 |
| ENG 816+2T>**C** | F: ACCACAACATGCAGATCTGGGC R: TCCCTCACGTATGGGCATAGG | Exon 6 Intron 6 | 147 |
| ENG 1195**delA** | F: GCTGTGAGGCAGAGGACG R: CAGCCTGCTCTCCCAAACAC | Exon 9a Intron 9a | 199 |
| ENG 1384**insT** | F: CTCAGCCCACACTTCCTCCT R: GCCACATCCTTGTCAGTGTC | Exon 10 Intron 10 | 243 |
|  |  |  |  |

^1^part of the human ß-globin gene was used as an internal control in each PCR-SSP and was amplified by

using the primers F: GGTTGGCCAATCTACTCCCAGG and R: GCTCACTCAGTGTGGCAAAG (540

bp PCR product).
