# Supplementary material for: Mutation analysis of "Endoglin" and "Activin receptor-like kinase" genes in German patients with hereditary hemorrhagic telangiectasia and the value of rapid genotyping using an allele-specific PCR-technique
Source: BMC Med Genet. 2009 Jun 9;10:53. doi: 10.1186/1471-2350-10-53 (PMC2701415; doi:10.1186/1471-2350-10-53)
Supplement: Additional file 4 — Table 4. Summary of mutations identified in the ENG gene. [file 1471-2350-10-53-S4.doc]

**Table 4. Summary of mutations identified in the *ENG* gene.**

__________________________________________________________________________

**Position** **Mutation** **Protein** **Type of** **Patient/** **Reference**

**(cDNA)** **change** **mutation** **family**

__________________________________________________________________________

----------------------------------------------------------------------------------------------------------------------------------------------------------------------------------------------------------------------------------------------------------------------------------------------------------------------------------------------------

Intron 3 c.360+1G>A p.Gly_Tyr120del splice mutation 21 S 26, 28, 31

_________________________________________________________________________________

Exon 4 c.392C>T p.Pro131Leu missense 23 S 3, 5, 34

_________________________________________________________________________________

Intron 6 c.816+2T>C unknown splice mutation 19 F^3^, 24 F^3^ 28, 31

_________________________________________________________________________________

Exon 9a c.1195delA p.Arg399fs deletion/fs 35 S 26

_________________________________________________________________________________

**Exon 10** **c.1384insT p.Gln462fs insertion/fs 11 S** **Novel mutation**

_________________________________________________________________________________

F^1-4^: family cases, S: single cases; mutation numbering based on cDNA sequences (NM_000118.1) with +1 as A of ATG codon; substitution (>), deletion (del), insertion (ins); references as given in the literature. Novel mutation is given in bold.
