# Supplementary material for: Mutation analysis of "Endoglin" and "Activin receptor-like kinase" genes in German patients with hereditary hemorrhagic telangiectasia and the value of rapid genotyping using an allele-specific PCR-technique
Source: BMC Med Genet. 2009 Jun 9;10:53. doi: 10.1186/1471-2350-10-53 (PMC2701415; doi:10.1186/1471-2350-10-53)
Supplement: Additional file 5 — Table 5. Polymorphisms in ENG. [file 1471-2350-10-53-S5.doc]

**Table 5. Polymorphisms in *ENG*.**

__________________________________________________________________________

**Position** **Polymorphism** **Affected** **Allelic** **Patient** **Reference**

**(cDNA)** **amino acid** **frequency**

__________________________________________________________________________

----------------------------------------------------------------------------------------------------------------------------------------------------------------------------------------------------------------------------------------------------------------------------------------------------------------------------------------------------

Exon 2 c.207G>A p.Leu69Leu 12x (29.3%) 02 S, 04 S, 06 S, 08 S, 6, 15, 40

10 S, 16 S, 17 S, 20 S,

28 F^3^, 31 F^4^, 34 S, 37 F^4^

________________________________________________________________________________

Exon 8 c.1029C>T p.Thr343Thr 7x (17%) 01 F^1^, 05 S, 07 S, 15, 40

30 S, 32 S, 39 S, 41 F^2^

_________________________________________________________________________________

Exon 10 c.1347A>G p.Pro458Pro 1x (2.4%) 33 F^4^ 31

_________________________________________________________________________________

#### Exon 13 c.1771G>A p.Ala591Thr 1x (2.4%) 29 S

---------------------------------------------------------------------------------------------------

Exon 13 c.1844C>T p.Ser615Leu 2x (4.8%) 03 S, 36 S _________________________________________________________________________________

*ENG* polymorphism numbering based on cDNA sequences (NM_000118.1); substitution (>); references as given in the literature.
