# Supplementary material for: Mutation analysis of "Endoglin" and "Activin receptor-like kinase" genes in German patients with hereditary hemorrhagic telangiectasia and the value of rapid genotyping using an allele-specific PCR-technique
Source: BMC Med Genet. 2009 Jun 9;10:53. doi: 10.1186/1471-2350-10-53 (PMC2701415; doi:10.1186/1471-2350-10-53)
Supplement: Additional file 6 — Table 6. Summary of mutations identified in the ACVRL1 gene. [file 1471-2350-10-53-S6.doc]

**Table 6. Summary of mutations identified in the *ACVRL1* gene.**

_________________________________________________________________________

**Position** **Mutation** **Protein** **Type of** **Patient** **Reference**

**(cDNA)** **change** **mutation** **no.**

__________________________________________________________________________

----------------------------------------------------------------------------------------------------------------------------------------------------------------------------------------------------------------------------------------------------------------------------------------------------------------------------------------------------

Exon 3 c.144_145insG p.Ala49fs insertion 12 F^2^, 13 F^2^ 38

---------------------------------------------------------------------------------------------------

Exon 3 c.200G>A p.Arg67Gln missense 38 S 18, 28, 31

_________________________________________________________________________________

Exon 5 c.540_541insA p.Asp181fs insertion 9 F^1^ 36

_________________________________________________________________________________

Exon 6 c.673_674delAG p.Ser225fs deletion/fs 14 S 35

---------------------------------------------------------------------------------------------------

Exon 6 c.696_698delCTC p.Ser233del in-frame deletion 18 S 18, 27, 31, 37, 40

_________________________________________________________________________________

Exon 8 c.1120C>T p.Arg374Trp missense 25 S, 27 S 2, 5, 32, 36, 39

---------------------------------------------------------------------------------------------------

Exon 8 c.1231C>T p.Arg411Trp missense 26 S 2, 17, 27, 28, 31, 36

---------------------------------------------------------------------------------------------------

Exon 8 c.1232G>A p.Arg411Gln missense 15 S 18, 19, 26, 28, 39

_________________________________________________________________________________

#### Exon 9 c.1346C>T p.Pro449Leu missense 40 S 36

---------------------------------------------------------------------------------------------------

Intron 9 c.1377+1G>A unknown splice mutation 22 S 20

_________________________________________________________________________________

F^1-4^ : family cases, S: single cases; mutation numbering based on cDNA sequences (NM_000118.1) with +1 as A of ATG codon; substitution (>), deletion (del), insertion (ins); references as to be seen in the literature part.
