# Supplementary material for: Mutation analysis of "Endoglin" and "Activin receptor-like kinase" genes in German patients with hereditary hemorrhagic telangiectasia and the value of rapid genotyping using an allele-specific PCR-technique
Source: BMC Med Genet. 2009 Jun 9;10:53. doi: 10.1186/1471-2350-10-53 (PMC2701415; doi:10.1186/1471-2350-10-53)
Supplement: Additional file 7 — Table 7. Genotype-phenotype correlation in HHT patients with confirmed mutation. [file 1471-2350-10-53-S7.doc]

**Table 7. Genotype-phenotype correlation in HHT patients with confirmed mutation.**

# Patient Sex Heredity Epistaxis Telangiectasia Organ Mutation Curacao

**no. manifestation criteria**

-----------------------------------------------------------------------------------------------------------------------------------------------

#11 S f x x x GI-tract, lung *ENG* 4

-----------------------------------------------------------------------------------------------------------------------------------------------

#19 F^3^ m x x x lung, brain ENG 4

------------------------------------------------------------------------------------------------------------------------------------- ----------

#21 S m x x x lung *ENG* 4

-----------------------------------------------------------------------------------------------------------------------------------------------

#23 S f x x x ---- *ENG* 2

-----------------------------------------------------------------------------------------------------------------------------------------------

#24 F^3^ m x x x lung *ENG*  4

-----------------------------------------------------------------------------------------------------------------------------------------------

#35 S m x x x GI-tract, lung *ENG* 4

-----------------------------------------------------------------------------------------------------------------------------------------------

-----------------------------------------------------------------------------------------------------------------------------------------------

#09 F^1^ f x x x ---- *ACVRL1* 3

-----------------------------------------------------------------------------------------------------------------------------------------------

#12 F^2^ f x x x GI-tract, liver, lung *ACVRL1* 4

-----------------------------------------------------------------------------------------------------------------------------------------------

#13 F^2^ m x x ---- ---- *ACVRL1* 2

-----------------------------------------------------------------------------------------------------------------------------------------------

#14 S f x x x liver *ACVRL1* 4

-----------------------------------------------------------------------------------------------------------------------------------------------

#15 S f x x x ---- *ACVRL1* 2

-----------------------------------------------------------------------------------------------------------------------------------------------

#18 S m x x x ---- *ACVRL1* 3

-----------------------------------------------------------------------------------------------------------------------------------------------

#22 S f x x x GI-tract *ACVRL1* 4

-----------------------------------------------------------------------------------------------------------------------------------------------

#25 S f x x x GI-tract *ACVRL1* 3

-----------------------------------------------------------------------------------------------------------------------------------------------

#26 S f x x x ---- *ACVRL1* 3

-----------------------------------------------------------------------------------------------------------------------------------------------

#27S f x x x GI-tract, liver *ACVRL1* 4

-----------------------------------------------------------------------------------------------------------------------------------------------

#38 S m x x x GI-tract, liver *ACVRL1* 3

-----------------------------------------------------------------------------------------------------------------------------------------------

#40 S m x x x ---- *ACVRL1* 3

­­­­­­­­­­­­­­­­­­­

Legend:

f = female, m = male, x = present

Curacao criteria: hereditary, epistaxis, telangiectasia, organ manifestation

(<2 criteria: HHT probable, >3 criteria: HHT manifest)
